# The relationship of remnant cholesterol and abdominal obesity in children: A cross-sectional study

**DOI:** 10.3389/fcvm.2022.929560

**Published:** 2022-07-27

**Authors:** Jishuang Tong, Xinggui Li, Xiaoyue Liang, Fang Tang, Yanling Ren, Guang Hao, Xin Peng, Sunqing Luo, Ye Feng, Daochao Huang, Li Zhao, Xiaohua Liang

**Affiliations:** ^1^Clinical Epidemiology and Biostatistics Department, Children's Hospital of Chongqing Medical University, Ministry of Education Key Laboratory of Child Development and Disorders, National Clinical Research Center for Child Health and Disorders, Key Laboratory of Pediatrics in Chongqing, China International Science and Technology Cooperation Center of Child Development and Critical Disorders, Chongqing, China; ^2^Shimian People's Hospital, Ya'an, China; ^3^Chongqing Medical and Pharmaceutical College, Chongqing, China; ^4^Department of Public Health and Preventive Medicine, School of Medicine, Jinan University, Guangzhou, China

**Keywords:** abdominal obesity, children, remnant cholesterol, urban-rural area, waist-to-height ratio

## Abstract

**Background:**

Previous studies found that remnant cholesterol (RC) is a risk factor for cardiovascular diseases and childhood obesity is associated with cardiometabolic diseases. This study aimed to explore the relationship between RC and abdominal obesity in children.

**Methods:**

A total of 5,959 children, aged 6−12 years old, were selected from a cross-sectional study in urban-rural areas of Chongqing, China. RC was calculated by total cholesterol (TC)—high-density lipoprotein (HDL-C) cholesterol—low-density lipoprotein (LDL-C) cholesterol and was divided into four groups by quartiles (Q1–Q4).

**Results:**

Compared to children with the lowest RC (Q1), children with higher RC had significantly higher odds of abdominal obesity (Q2: OR = 1.450, 95% CI: 1.131−1.859, *p* < 0.05; Q3: OR = 2.127, 95% CI: 1.632−2.772, *p* < 0.001; Q4: OR = 2.386, 95% CI: 1.819−3.130, *p* < 0.001). In the stratified analyses by urban-rural areas, the odds ratios were greater in rural areas (Q2: OR = 2.228, 95% CI: 1.572−3.160, *p* < 0.001; Q3: OR = 3.668, 95% CI: 2.191−6.140, *p* < 0.001; Q4: OR = 6.490, 95% CI: 2.271−18.551, *p* < 0.001) than in urban areas (Q2: OR = 1.644, 95% CI: 1.192−2.266, *p* < 0.05; Q3: OR = 2.266, 95% CI: 1.667−3.082, *p* < 0.001; Q4: OR = 2.711, 95% CI: 2.005−3.665, *p* < 0.001).

**Conclusions:**

Our study found that RC was positively correlated with abdominal obesity in children, and this association was higher for children living in rural areas.

## Introduction

Remnant cholesterol (RC) is the amount of cholesterol in triglyceride (TC)-rich lipoproteins produced by the liver and the intestine. It can also be considered as the sum of all non-low-density lipoprotein (LDL) and non-high-density lipoprotein (HDL) cholesterol levels (1). RC is associated with higher cardiometabolic risk and contributes to the residual risk after controlling LDL cholesterol (2, 3). In recent years, the prevalence of childhood obesity, especially abdominal obesity, has increased dramatically worldwide (4–7). Childhood obesity is a primary contributor to the development of cardiometabolic diseases, including dyslipidemia (8, 9). Therefore, it is particularly important to identify biochemical markers in children to help identify high-risk children early and conduct interventions in advance. While traditional lipid markers are not usually elevated in children with obesity (10), RC is predictive of coronary artery stenosis in patients with normal serum TC levels (11). Previous studies also showed that RC is associated with obesity, triglycerides, and systolic blood pressure (BP) and could mediate the transition from obesity to ischemic cardiomyopathy (12, 13). Further, apolipoprotein B48 (specific to RC) is more strongly associated with central adiposity in obese prepubertal children, compared to classical lipid marker LDL-C or total cholesterol (TC) (14). Therefore, RC may be more representative of early cardiovascular risk compared to LDL. However, there are limited data on children and adolescents, but there are no studies on Asian children.

This study hypothesized that RC is correlated with abdominal obesity in children and an urban-rural difference may exist. A large cohort from Chongqing, China was used to test the hypothesis. The results could provide a basis for the prevention of early dyslipidemia in children with obesity and to reduce the cardiovascular risk in adulthood.

## Methods

### Subjects

**Figure 1:**
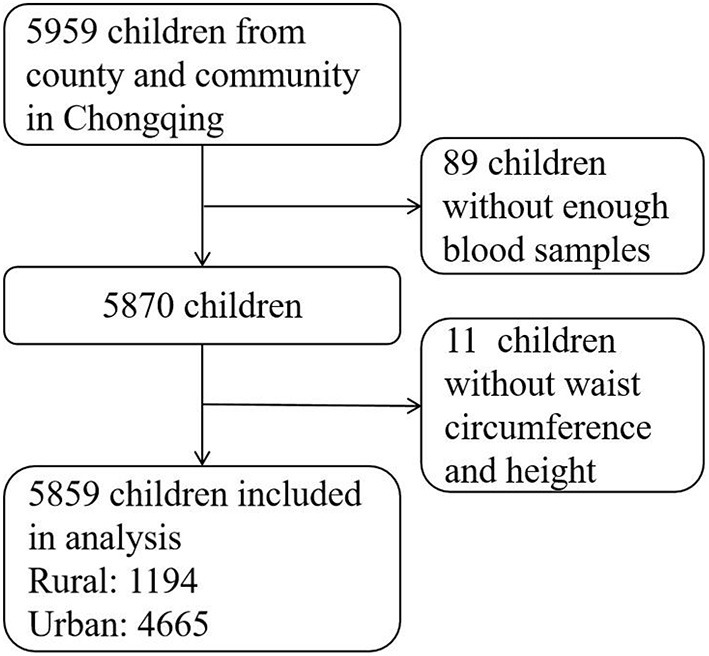
The flow chart of participants including.

The Chongqing Children's Health Cohort was selected through a two-stage stratified (county and community) sampling. Details of participant recruitment and questionnaire collection were described in a previously published article (15). The baseline data collected in 2014 was used to assess the relationship between RC and abdominal obesity. Participants who met the following inclusion criteria were included in this study: (1) aged 6–12 years old, (2) living in the target area for more than 6 months, (3) free of serious diseases, such as cardiovascular diseases, nephropathy, and cancer, and, (4) both participants and their parents/guardians gave informed consent, and (5) gave permission to collect blood samples. Finally, 5,859 samples were analyzed after excluding participants without enough blood samples to check serum lipid levels (*n* = 89) or without data on waist circumference and height (*n* = 11) (shown in Figure 1). The Medical Research Ethics Committee of the Research Division of the Children's Hospital of Chongqing Medical University gave its approval for the study, approval number (No. 2019-86).

### Demographic variables and dietary intake

Demographic data included age, gender, and residing in rural/urban areas. Socioeconomic status (SES) including parents' occupation, fathers' education level, household income, living situation, people living with a child, and medical insurance were collected. Parental education level was represented by a 4-point scale (≤ 9, 10–12, 13–15, and >15 years) (16, 17). Data on birth weight, breastfeeding, and family history of obesity were also collected. Dietary intakes were collected using a quantitative food frequency questionnaire, as previously described in detail (18).

### Physical examination

Physical examination was conducted by trained pediatric nurses, as previously described (19). In brief, height and weight were measured using a mobile medical ultrasonic machine (model WS-H300D). Body mass index (BMI) was calculated by weight/height^2^ (kg/m^2^). Waist circumference was measured two times at the center of the umbilicus, and the average value was taken to calculate the waist-to-height ratio (WHtR) as a measure of abdominal obesity.

Blood pressure (BP) and heart rate were measured on three different occasions using an OMRON arm-mounted electronic sphygmomanometer (HEM7051). Children were diagnosed with hypertension if all three BP measurements met the criteria for hypertension according to the diagnostic criteria for Chinese children (20).

### Biochemical indexes

Blood samples were taken after at least 12 h of fasting for the measurement of biochemical indexes, including fasting blood glucose (FBG), TC, TG, high-density lipoprotein cholesterol (HDL-C), and low-density lipoprotein cholesterol (LDL-C). TC was measured by the Cholesterol Determination - Peroxidase and 4-Aminoantipyrine substrate method. The mixture of cholesteryl esters and free cholesterol was reacted with cholesteryl esterase and cholesterol oxidase to produce hydrogen peroxide, which reacted with 4-aminoantipyrine and produced quinone imine compounds. HDL cholesterol was measured by the direct peroxidase clearance method in two steps. In the first step, reagent one (buffer 2-ethanesulfonic acid and surfactant 1) and serum lipid compositions (celiac particles, very-low-density lipoproteins (VLDL), and LDL) were reacted to expose the cholesterol in the serum. After cholesterol esterase and cholesterol oxidase catalyzed the cholesterol, hydrogen peroxide was produced. In the second step, similarly, another surfactant was used to expose the cholesterol in HDL, which would react with cholesterol enzymes for chromogenic reaction, and the shade of color was proportional to the amount of HDL cholesterol. LDL cholesterol was measured by the direct surfactant removal method. Except for LDL, surfactant 1 was used to alter the structure of lipoproteins (chylomicron, VLDL, HDL, etc.), and the metabolite would react with cholesterol esterases and cholesterol oxidases. As a result, all lipoproteins, except LDL, were eliminated. Then, a chromogenic reaction of LDL occurred with surfactant 2. The shade of color was proportional to the amount of LDL cholesterol. The details were described in a previous study (15), and RC was calculated by TC—HDL-C—LDL-C and was divided into four groups by quartiles (Q1–Q4).

### Diagnostic criteria

A threshold of WHtR ≥0.480 for boys and WHtR ≥ 0.456 for girls were identified for abdominal obesity in childhood (21).

### Statistical analyses

Normally distributed continuous variables were expressed as mean ± standard deviation, and the differences between rural and urban areas were assessed with Student's *t*-test. Skewed distribution continuous variables (e.g., diet) were reported as medians (interquartile range), and the Mann–Whitney U test was used for comparison between the two groups. The Chi-square test was used to compare categorical variables. A linear regression model was used to evaluate the association between RC and WHtR, and a logistic regression model was used to assess the relationship between abdominal obesity and quartile range increase of RC.

Statistical analysis was performed using SAS 9.4 software (Copyright^©^ 2016 SAS Institute Inc., Cary, NC, USA). A *p-*value <0.05 was considered statistically significant.

## Results

### General characteristics

The demographic characteristics of the participants are presented in Table 1. The mean age was 9.14 ± 1.76 years, and 52.3% (3063/5859) were boys. There are differences in the characteristics of children living in urban and rural areas (Table 1). Therefore, subgroup analyses of the urban-rural areas were performed in this study.

**Table 1:** The demographic characteristics of children in rural and urban areas.

**Variables**	**Total**	**Rural**	**Urban**	***P***
Anthropometric variables				
Male, *n* (%)	3,063 (52.3%)	656 (54.9%)	2,407 (51.6%)	0.015
Female, *n* (%)	2,796 (47.7%)	538 (45.1%)	2,258 (48.4%)	
Age, year	9.11 (7.68, 10.75)	9.67 (7.96, 11.02)	9.00 (7.61, 10.64)	<0.001
Height, cm	135.00 (126.50, 144.00)	136.50 (127.00, 146.00)	134.50 (126.38, 143.50)	<0.001
Weight, kg	31.10 (25.50, 39.40)	34.00 (26.00, 45.00)	30.70 (25.60, 38.00)	<0.001
Birth weight, g	6.70 (6.00, 7.50)	6.70 (6.00, 7.60)	6.70 (6.00, 7.40)	<0.001
Waist, cm	58.00 (53.00, 65.00)	59.00 (53.00, 71.00)	58.00 (53.00, 64.00)	<0.001
Heart rate, n/min	95.00 (87.00, 104.00)	96.00 (88.00, 105.00)	94.00 (86.00, 103.00)	<0.001
BMI, kg/m^2^	17.03 (15.50, 19.61)	17.75 (15.50, 22.81)	16.88 (15.50, 19.02)	<0.001
Waist-to-height ratio	0.43 (0.40, 0.47)	0.44 (0.40, 0.50)	0.43 (0.40, 0.47)	0.036
SBP, mmHg	103.30 (96.70, 110.30)	108.00 (99.30, 117.00)	102.30 (96.00, 108.70)	<0.001
DBP, mmHg	63.00 (58.30, 68.30)	65.70 (60.70, 71.70)	62.30 (58.00, 67.30)	<0.001
Hypertension, *n* (%)				
Yes	761 (13.0%)	352 (29.5%)	409 (8.8%)	<0.001
No	5,098 (87.0%)	841 (70.5%)	4,255 (91.2%)	
Abdominal obesity, *n* (%)				
Yes	1,596 (27.3%)	426 (35.6%)	1,170 (25.2%)	<0.001
No	4,249 (72.7%)	768 (64.4%)	3,481 (74.8%)	
Serum biochemical index				
FBG, mmol/L	4.06 (3.83, 4.58)	4.90 (4.61, 5.18)	3.94 (3.71, 4.17)	<0.001
TC, mmol/L	3.48 (3.09, 3.93)	4.06 (3.67, 4.47)	3.32 (2.99, 3.69)	<0.001
TG, mmol/L	0.83 (0.60, 1.18)	0.88 (0.66, 1.20)	0.81 (0.58, 1.16)	0.137
HDL-C, mmol/L	1.19 (1.04, 1.39)	1.30 (1.17, 1.49)	1.16 (1.01, 1.35)	<0.001
LDL-C, mmol/L	1.66 (1.38, 2.04)	2.30 (1.96, 2.65)	1.53 (1.31, 1.79)	<0.001
RC, mmol/L				
First quartile	0.35 (0.28, 0.39)	0.33 (0.26, 0.37)	0.36 (0.30, 0.39)	<0.001
Second quartile	0.48 (0.45, 0.51)	0.47 (0.44, 0.51)	0.49 (0.46, 0.52)	
Third quartile	0.60 (0.57, 0.63)	0.58 (0.56, 0.62)	0.60 (0.57, 0.63)	
Fourth quartile	0.77 (0.71, 0.86)	0.72 (0.69, 0.89)	0.77 (0.71, 0.86)	
TC, mmol/L				
First quartile	3.34 (2.84, 3.96)	4.02 (3.65, 4.40)	2.89 (2.59, 3.23)	<0.001
Second quartile	3.31 (2.94, 3.75)	4.05 (3.69, 4.48)	3.12 (2.84, 3.45)	
Third quartile	3.39 (3.11, 3.74)	4.24 (3.74, 4.75)	3.34 (3.09, 3.66)	
Fourth quartile	3.67 (3.35, 4.02)	4.21 (3.51, 5.28)	3.65 (3.35, 4.02)	
LDL-C, mmol/L				
First quartile	1.70 (1.28, 2.30)	2.36 (2.03, 2.69)	1.34 (1.12, 1.55)	<0.001
Second quartile	1.57 (1.31, 1.92)	2.19 (1.86, 2.62)	1.44 (1.25, 1.68)	
Third quartile	1.58 (1.38, 1.85)	2.30 (1.82, 2.77)	1.56 (1.36, 1.79)	
Fourth quartile	1.71 (1.45, 1.96)	2.07 (1.69, 2.52)	1.71 (1.45, 1.95)	
HDL-C, mmol/L				
First quartile	1.23 (1.07, 1.44)	1.27 (1.13, 1.49)	1.20 (1.04, 1.39)	<0.001
Second quartile	1.21 (1.06, 1.39)	1.32 (1.19, 1.50)	1.17 (1.01, 1.36)	
Third quartile	1.17 (1.03, 1.38)	1.33 (1.23, 1.47)	1.15 (1.02, 1.35)	
Fourth quartile	1.14 (1.00, 1.32)	1.36 (1.22, 1.48)	1.14 (1.00, 1.31)	
TG, mmol/L				
First quartile	0.69 (0.52, 0.94)	0.80 (0.61, 1.06)	0.61 (0.45, 0.84)	<0.001
Second quartile	0.77 (0.58, 1.03)	0.93 (0.72, 1.27)	0.70 (0.53, 0.95)	
Third quartile	0.86 (0.64, 1.18)	1.20 (0.89, 1.70)	0.83 (0.63, 1.13)	
Fourth quartile	1.07 (0.77, 1.54)	1.33 (0.91, 2.40)	1.07 (0.77, 1.53)	
Blood-cell composition				
HGB, g/l	127.00 (121.00, 133.00)	136.00130.00, 141.00)	125.00 (120.00, 130.00)	<0.001
Dietary intakes				
Cereals and potatoes	150.00 (100.00, 250.00)	150.00 (100.00, 252.92)	150.00 (100.00, 250.00)	<0.001
Vegetables	150.00 (100.00, 250.00)	164.29 (100.00, 250.00)	150.00 (100.00, 250.00)	0.590
Fruit	150.00 (71.43, 250.00)	142.86 (71.43, 250.00)	150.00 (71.43, 250.00)	0.502
Red meat	100.00 (50.00, 15.00)	100.00 (50.00, 150.00)	100.00 (50.00, 150.00)	0.536
Poultry	35.71 (14.29, 71.43)	33.33 (14.29, 71.43)	35.71 (14.29, 71.43)	0.003
Fish	16.67 (6.85, 35.71)	16.67 (5.00, 41.25)	16.67 (7.14, 35.71)	0.935
Eggs	50.00 (21.43, 100.00)	50.00 (14.29, 91.67)	50.00 (21.43, 100.00)	0.252
Milk	250.00 (200.00, 250.00)	250.00 (142.86, 250.00)	250.00 (190.00, 260.00)	<0.001
Bean food	33.33 (14.29, 71.43)	33.33 (14.29, 71.43)	28.57 (14.29, 71.43)	<0.001
Nuts	14.29 (3.33, 35.71)	13.33 (1.67, 35.71)	14.29 (3.33, 35.71)	0.443
Mushrooms and algae food	10.00 (3.33, 33.33)	8.33 (1.67, 35.54)	14.29 (3.33, 33.33)	0.307
Oils	50.00 (50.00, 100.00)	50.00 (42.86, 100.00)	50.00 (50.00, 100.00)	0.439
Pickle	6.67 (0.83, 14.29)	7.14 (0.83, 21.43)	5.00 (0.68, 14.29)	<0.001
Nutritional supplements	0.00 (0.00, 1.78)	0.00 (0.00, 7.09)	0.00 (0.00, 1.67)	<0.001
Beverage	14.29 (1.37, 35.71)	16.67 (0.82, 64.29)	14.29 (1.10, 33.33)	<0.001
Father's occupation, *n *(%)				
Manager	406 (7.1%)	95 (8.1%)	311 (6.9%)	0.666
Worker	1,717 (30.2%)	360 (30.7%)	1,357 (30.0%)	
Technicist/Researcher	323 (5.7%)	26 (2.2%)	297 (6.6%)	
Farmer	1,760 (30.9%)	448 (38.2%)	1,312 (29.1%)	
Others	1,484 (26.1%)	244 (20.8%)	1,240 (27.4%)	
Mother's occupation, *n* (%)				
Manager	252 (4.4%)	63 (5.5%)	189 (4.2%)	<0.001
Worker	1,582 (27.9%)	292 (25.3%)	1,290 (28.5%)	
Technicist/Researcher	117 (2.1%)	14 (1.2%)	103 (2.3%)	
Farmer	1,927 (34.0%)	313 (27.2%)	1,614 (35.7%)	
Others	1,794 (31.6%)	470 (40.8%)	1,324 (29.3%)	
Father's education level, *n* (%)				
~9	2,410 (42.4%)	710 (60.8%)	1,700 (37.7%)	<0.001
~12	2,527 (44.5%)	345 (29.6%)	2,182 (48.4%)	
~15	682 (12.0%)	105 (9.0%)	577 (12.8%)	
>15	59 (1.0%)	7 (0.6%)	52 (1.2%)	
Income, RMB, *n* (%)				
~500	175 (3.8%)	108 (9.5%)	67 (1.9%)	<0.001
~1,000	351 (7.5%)	165 (14.5%)	186 (5.3%)	
~2,000	757 (16.3%)	256 (22.5%)	501 (14.2%)	
~3,000	1,086 (23.3%)	266 (23.4%)	820 (23.3%)	
>3,000	2,288 (49.1%)	343 (30.1%)	1,945 (55.3%)	
People live with child, *n* (%)				
1	184 (4.0%)	67 (6.0%)	117 (3.3%)	0.162
2–3	2,581 (55.6%)	542 (48.2%)	2,039 (58.0%)	
4	1,876 (40.4%)	514 (45.8%)	1,362 (38.7%)	
Medical insurance				
Yes	4,137 (88.5%)	901 (79.0%)	3,236 (91.6%)	2.597
No	538 (11.5%)	240 (21.0%)	298 (8.4%)	
Living situation, *n* (%)				
Live with parents	3,458 (73.8%)	751 (65.6%)	2,707 (76.4%)	0.032
Live with grandparents	605 (12.9%)	204 (17.8%)	401 (11.3%)	
Live with father or mother	444 (9.5%)	143 (12.5%)	301 (8.5%)	
Others	178 (3.8%)	46 (4.0%)	132 (3.7%)	
Breastfeeding, month, *n* (%)				
0–3	1,320 (28.5%)	219 (19.6%)	1,101 (31.4%)	<0.001
4–10	2,276 (49.2%)	511 (45.6%)	1,765 (50.4%)	
>10	1,030 (22.3%)	391 (34.9%)	639 (18.2%)	
Father with obesity, *n* (%)				
Yes	829 (17.8%)	215 (18.9%)	614 (17.5%)	0.582
No	3,819 (82.2%)	921 (81.1%)	2,898 (82.5%)	
Mother with obesity, *n* (%)				
Yes	505 (10.9%)	151 (13.3%)	354 (10.1%)	0.004
No	4,145 (89.1%)	983 (86.7%)	3,162 (89.9%)	

*SBP, systolic blood pressure; DBP, systolic blood pressure; BMI, body mass index; FBG, fasting blood glucose; TC, total cholesterol; TG, triglyceride; HDL-C, high-density lipoprotein cholesterol; LDL-C, low-density lipoprotein cholesterol; HGB, hemoglobin*.

### Prevalence of abdominal obesity

**Figure 2:**
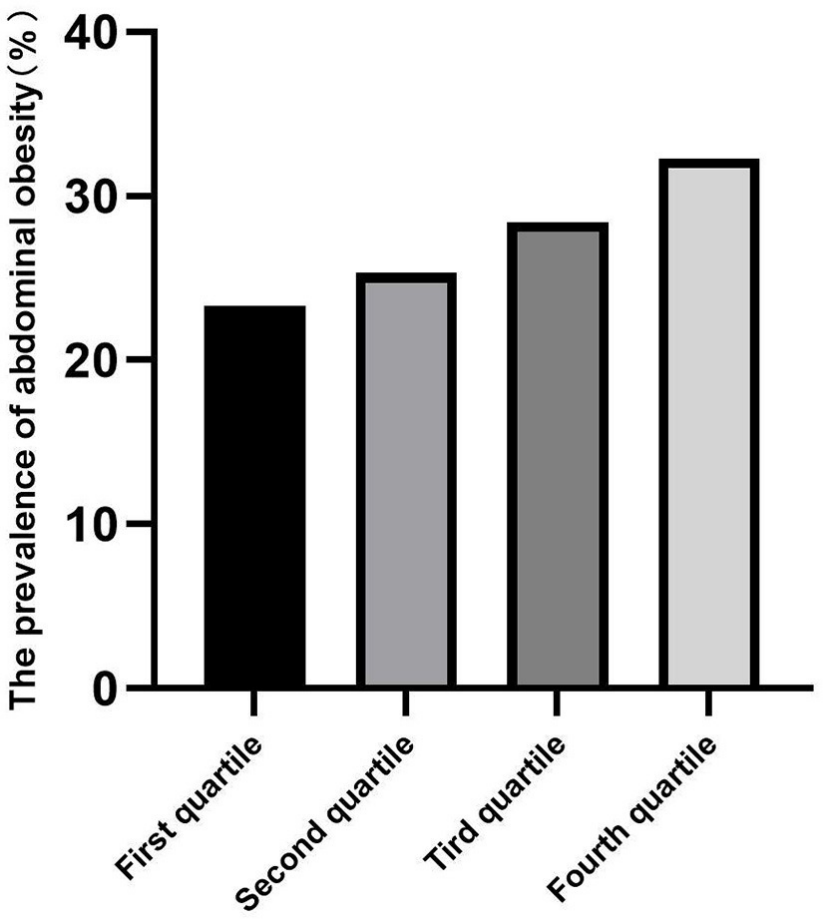
The prevalence of abdominal obesity among remnant cholesterol (RC) quartiles in total participants.

**Figure 3:**
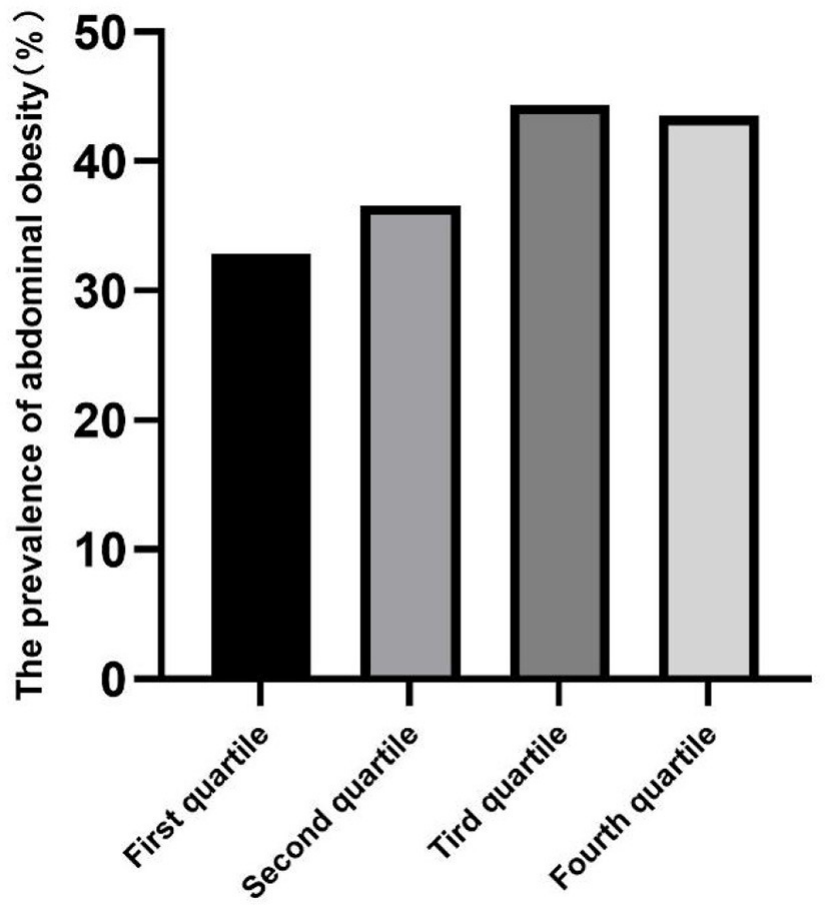
The prevalence of abdominal obesity among remnant cholesterol (RC) quartiles in rural.

**Figure 4:**
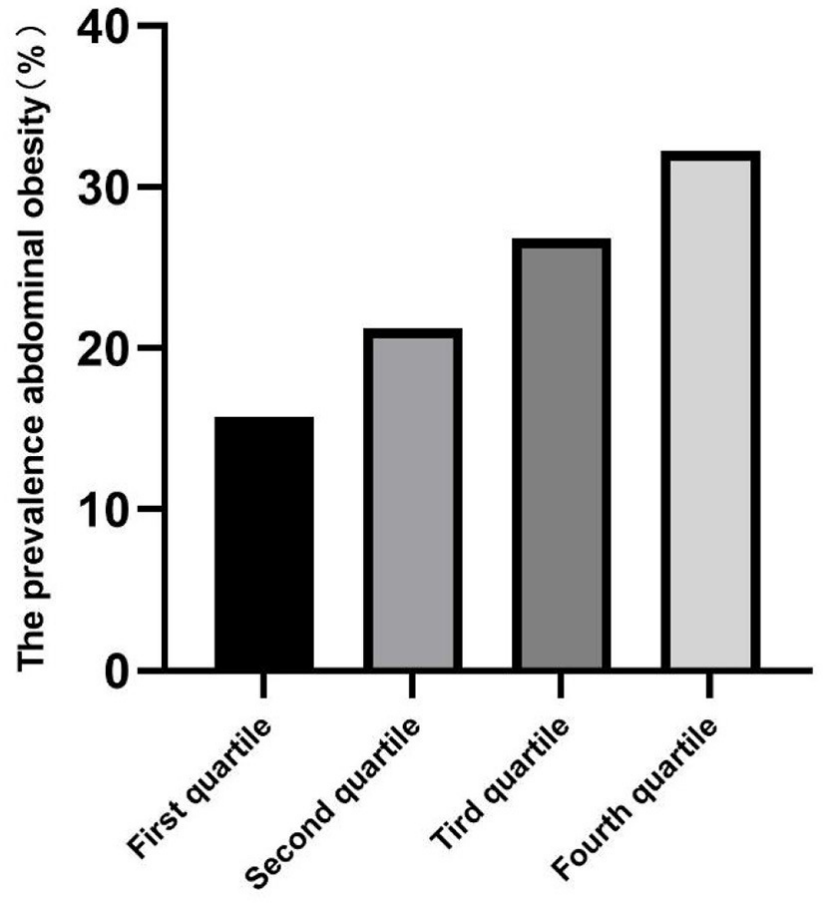
The prevalence of abdominal obesity among remnant cholesterol (RC) quartiles in urban.

The prevalence of abdominal obesity was higher in children with increased total RC, and the RC of children living in urban areas was higher than those living in rural areas (all *p* < 0.05) (Table 2). The prevalence of abdominal obesity increased with increasing RC quartiles in the total, urban, and rural areas (Figures 2–4). Additionally, the children with the highest RC had the highest prevalence of abdominal obesity (*p* for trend <0.05), which was found in both urban and rural areas.

**Table 2:** The prevalence of children with abdominal obesity aged 6–12 years.

**Variables**	**Prevalence**	***X* ^2^**	***P***	**P_trend_**
Total: RC
First quartile (ref)	23.3% (331/1,418)	33.545	<0.001	<0.001
Second quartile	25.3% (398/1,574)			
Third quartile	28.4% (399/1,406)			
Fourth quartile	32.3% (468/1,447)			
Rural: RC
First quartile (ref)	32.9% (207/629)	6.975	0.073	0.02
Second quartile	36.6% (152/415)			
Third quartile	44.4% (56/126)			
Fourth quartile	43.5% (10/23)			
Urban: RC
First quartile (ref)	15.7% (124/789)	88.708	<0.001	<0.001
Second quartile	21.2% (245/1,158)			
Third quartile	26.8% (343/1,280)			
Fourth quartile	32.2% (458/1,423)			

*RC, remnant cholesterol*.

### The univariate analysis of RC

Compared to the normal-weight children, the RC levels were higher in children with abdominal obesity, and the difference was significant both in urban and rural areas (all *p* < 0.001, in Table 3).

**Table 3:** The univariate analysis of RC.

**Variables**	**RC: P**_50_ **(P**_25_**, P**_75_**)**	**Z**	**P**
Total, abdominal obesity
Yes	0.57 (0.44, 0.70)	−0.606	P <0.01
No	0.53 (0.42, 0.66)		
Rural, abdominal obesity
Yes	0.42 (0.35, 0.50)	−4.979	P <0.01
No	0.40 (0.31, 0.48)		
Urban, abdominal obesity
Yes	0.63 (0.52, 0.74)	−9.768	P <0.01
No	0.56 (0.45, 0.69)		

*RC, remnant cholesterol*.

### The association between RC and WHtR

A linear regression model was used to examine the relationship between RC and WHtR in Table 4. After adjusting for other covariates in model 5, RC was significantly positively associated with WHtR (β = 0.021, 95% CI: 0.013−0.028, *p* < 0.05). In the subgroup analyses, the RC level was significantly positively associated with WHtR in children living in both urban (β = 0.019, 95% CI: 0.013−0.026, *p* < 0.05) and rural (β = 0.030, 95% CI: 0.009−0.053, *p* < 0.05) areas.

**Table 4:** The linear association between remnant cholesterol (RC) and waist-to-height ratio (WHtR).

**Models**	**Total**			**In rural**			**In urban**		
	**β (95%CI)**	**SE**	**P**	**β (95%CI)**	**SE**	***P***	**β (95%CI)**	**SE**	***P***
Model 1	0.016 (0.008, 0.023)	0.004	<0.01	0.031 (0.007, 0.055)	0.012	0.013	0.029 (0.020, 0.037)	0.004	<0.01
Model 2	0.020 (0.012, 0.027)	0.004	<0.01	0.027 (0.004, 0.051)	0.012	0.024	0.027 (0.019, 0.036)	0.004	<0.01
Model 3	0.022 (0.015, 0.029)	0.004	<0.01	0.029 (0.007, 0.054)	0.011	0.011	0.021 (0.014, 0.029)	0.004	<0.01
Model 4	0.022 (0.016, 0.030)	0.004	<0.01	0.033 (0.010, 0.058)	0.011	0.004	0.020 (0.013, 0.028)	0.004	<0.01
Model 5	0.021 (0.013, 0.028)	0.004	<0.01	0.030 (0.009, 0.053)	0.011	0.006	0.019 (0.013, 0.026)	0.004	<0.01

*Model 1: crude.*

*Model 2: adjusted variables in model 1, age, gender, and birth weight.*

*Model 3: adjusted variables in model 2, SBP, DBP, HGB, and heart rate.*

*Model 4: adjusted variables in model 3, vegetables, red meat, fish, eggs, and milk.*

*Model 5: adjusted variables in model 4, father's occupation, mother's occupation, father's education level, income, region, people living with the child, breastfeeding, father with obesity, and mother with obesity*.

### The association between quartile range increase of RC and abdominal obesity

A logistic regression model was used to investigate the relationship between RC by quartiles and abdominal obesity in Table 5. After controlling for other covariates in model 5, compared to children with the lowest RC (Q1), children with higher RC had significantly higher odds of abdominal obesity (Q2: OR = 1.450, 95% CI: 1.131−1.859, *p* < 0.05; Q3: OR = 2.127, 95% CI: 1.632−2.772, *p* < 0.001; Q4: OR = 2.386, 95% CI: 1.819−3.130, *p* < 0.001). In the stratified analyses by urban-rural areas, the odds ratios were greater in rural areas (Q2: OR = 2.228, 95% CI: 1.572−3.160, *p* < 0.001; Q3: OR = 3.668, 95% CI: 2.191−6.140, *p* < 0.001; Q4: OR = 6.490, 95% CI: 2.271−18.551, *p* < 0.001) than in urban areas (Q2: OR =1.644, 95% CI: 1.192−2.266, *p* < 0.05; Q3: OR = 2.266, 95% CI: 1.667−3.082, *p* < 0.001; Q4: OR = 2.711, 95% CI: 2.005−3.665, *p* < 0.001).

**Table 5:** The logistic regression analysis of quartile range increase of the RC level with abdominal obesity.

**Models**	**Total**		**In rural**		**In urban**	
	**OR (95%CI)**	**P**	**OR (95%CI)**	**P**	**OR (95%CI)**	**P**
Model 1
Q1, reference
Q2	1.213 (0.966, 1.523)	0.096	1.226 (0.928, 1.619)	0.151	1.670 (1.227, 2.273)	0.001
Q3	1.529 (1.215, 1.924)	<0.001	1.620 (1.070, 2.452)	0.023	2.433 (1.812, 3.267)	<0.001
Q4	1.613 (1.287, 2.023)	<0.001	1.419 (0.596, 3.381)	0.43	2.971 (2.229, 3.961)	<0.001
Model 2
Q1, reference
Q2	1.292 (1.023, 1.632)	0.031	1.223 (0.918, 1.630)	0.17	1.682 (1.233, 2.293)	0.001
Q3	1.682 (1.328, 2.130)	<0.001	1.666 (1.085, 2.559)	0.02	2.429 (1.806, 3.268)	<0.001
Q4	1.802 (1.427, 2.275)	<0.001	1.541 (0.631, 3.763)	0.342	2.987 (2.235, 3.991)	<0.001
Model 3
Q1, reference
Q2	1.374 (1.083, 1.744)	0.009	2.151 (1.537, 3.012)	<0.001	1.615 (1.177, 2.217)	0.003
Q3	1.808 (1.419, 2.303)	<0.001	3.519 (2.131, 5.811)	<0.001	2.218 (1.638, 3.004)	<0.001
Q4	1.915 (1.507, 2.433)	<0.001	6.030 (2.165, 16.798)	0.001	2.674 (1.987, 3.599)	<0.001
Model 4
Q1, reference
Q2	1.376 (1.084, 1.748)	0.009	2.205 (1.565, 3.108)	<0.001	1.618 (1.178, 2.222)	0.003
Q3	1.824 (1.430, 2.327)	<0.001	3.710 (2.227, 6.180)	<0.001	2.238 (1.652, 3.032)	<0.001
Q4	1.934 (1.520, 2.461)	<0.001	6.416 (2.297, 17.923)	<0.001	2.698 (2.004, 3.634)	<0.001
Model 5
Q1, reference
Q2	1.450 (1.131, 1.859)	0.003	2.228 (1.572, 3.160)	<0.001	1.644 (1.192, 2.266)	0.002
Q3	2.127 (1.632, 2.772)	<0.001	3.668 (2.191, 6.140)	<0.001	2.266 (1.667, 3.082)	<0.001
Q4	2.386 (1.819, 3.130)	<0.001	6.490 (2.271, 18.551)	<0.001	2.711 (2.005, 3.665)	<0.001

*Q1: First quartile of RC; Q2: Second quartile of RC; Q3: Third quartile of RC; Q4: Fourth quartile of RC, remnant cholestero*

*Model 1: crude.*

*Model 2: adjusted model 1, age, sex, and birth weight.*

*Model 3: adjusted model 2, DBP, HGB, and heart rate.*

*Model 4: adjusted model 3, vegetables, fruit, red meat, fish, eggs, milk, nuts, mushrooms and algae food, oils, and nutritional supplements.*

*Model 5: adjusted model 4, mother's occupation, father's education level, income, region, people living with the child, father with obesity, and mother with obesity*.

## Discussion

Our study showed a significant positive association between RC and abdominal obesity in children. Children living in urban areas had a higher level of RC than children living in rural areas. However, the prevalence of abdominal obesity among children in rural areas was higher than that in urban areas. The association between RC and abdominal obesity was higher for children living in rural areas.

Similarly, previous studies found that, both in children and adults, RC was positively correlated with waist circumference and BMI (2, 13, 22). In our study, the prevalence of abdominal obesity in children increased with the increase in the RC quartile range, which was consistent with previous studies. In a Danish study in adults, BMI increased with progressively higher RC concentrations in four subgroups. The median RC level in this study was higher than that observed in a previous study. This may be due to the differences in lipid levels between adults and children, and RC concentrations in this study were both calculated and measured (22). In addition, evidence from Di Costanzo et al. proved that the prevalence of overweight/obesity and BMI were gradually increased by the RC tertiles, and the RC level was significantly and positively correlated with BMI and waist circumference (23); besides, Varbo et al. found that BMI also increased with nonfasting RC tertile range increase (24). Moreover, our study and previous studies confirmed that the obese group had higher levels of RC than the normal weight group, both in adults and children (13, 25, 26). However, this study added new reliable evidence from a large sample size of urban-rural childhood participants. Since most of the fat in children is deposited in the abdomen, children who were not obese/overweight according to BMI may be abdominally obese, which was more common than generalized obesity (27). Interestingly, our study found that the association between RC and abdominal obesity was higher for children living in rural areas. This may be explained by the different lifestyle and dietary intake patterns.

The dietary structure of children had changed greatly during the past decades, and accompanying it, the incidence of childhood obesity had increased dramatically (7, 28, 29). Hepatic absorption and metabolism of cholesterol in celiac residues was influenced by the type of fat in the diet and the composition of fatty acid. A saturated fat-rich diet reduces the excretion of residual cholesterol from celiac disease (30). Nutritional excess and central obesity exacerbated dietary lipid intolerance and impaired metabolism of celiac residues, resulting in elevated plasma concentrations of TC-rich lipoprotein residues (31–33).

Remnant cholesterol (RC) had been found as an independent risk factor for cardiovascular disease, similar to LDL, and the RC in the bloodstream passed through the endothelium and collected within the arterial wall, where it was absorbed by macrophages and smooth muscle cells, forming foam cells that eventually became part of the atherosclerotic plaque (34). Atherosclerosis due to RC was primarily driven by RC concentrations in the bloodstream, independent of the cause of increased residual cholesterol, such as due to overweight and obesity (22). Individuals with obesity have higher RC concentrations. However, the etiological mechanism of increased RC was not clearly explained, such as whether and how overweight and obesity increased RC (22). Importantly, due to the complexity of lipid metabolism and the mechanisms of gene-environment interactions in metabolic diseases, previous studies found that the genetic variants with the greatest impact on RC concentrations were found at a key point of lipoprotein metabolisms, such as apolipoprotein E (APOE), apolipoprotein C3 (APOC3), and apolipoprotein C2 (APOC2) genes encoding apolipoproteins and genes encoding enzymes involved in residue degradation, such as lipoprotein lipase (LPL) (1).

Fasting RC was not only a risk factor for cardiovascular diseases such as atherosclerosis and myocardial infarction but was also associated with diabetes, kidney disease, and carotid artery intima-media thickness in adolescents (22, 23, 35–37). Moreover, it was a useful predictor of postprandial hyperlipidemia (38, 39). On the one hand, our results added evidence that fasting RC was positively correlated with obesity. On the other hand, postprandial RC was also found to be associated with waist-to-hip ratio (WHR) in adults, independent of BMI, and the increased risk of IHD due to obesity was mediated in part by non-fasting RC (12, 26). Therefore, the study of non-fasting RC will also be an important focus for us in the future. Further, studies showed that elevated non-fasting RC was causally associated with mild inflammation and IHD and that recent epidemiological data strongly supported RC in the non-fasting state as a contributor to residual risk (3, 24). More studies are required to investigate the roles of fasting and non-fasting RC in cardiovascular outcomes.

### Strengths and limitation

Obesity in children is mainly subcutaneous fat, so the use of abdominal obesity indicators is more appropriate than using general obesity diagnosed by BMI (40). Our study was the first study to examine the relationship between RC and abdominal obesity in a large Chinese cohort.

There are several limitations to this study. First, the RC calculated from the standard lipid profile as TC minus LDL-C minus HDL-C is different from the RC measured by nuclear magnetic resonance (41), as there was no uniform clinical method for measuring RC. Second, the causal relationship cannot be explained by a cross-sectional study. Third, the study did not exclude children with fatty liver disease and/or atherosclerosis and did not collect information on background therapy in this study. Therefore, future studies are needed to investigate the causal association between RC and abdominal obesity and potential etiological mechanisms.

## Conclusion

In our study, RC was positively associated with childhood abdominal obesity both in urban and rural areas. Moreover, the association was higher for children living in rural areas. Our findings provided a theoretical basis for cardiovascular prevention in children with abdominal obesity. Further cohort studies are needed to confirm the causal relationship between RC and abdominal obesity in children.

## Data availability statement

The original contributions presented in the study are included in the article/supplementary material, further inquiries can be directed to the corresponding author/s.

## Ethics statement

The studies involving human participants were reviewed and approved by the Medical Research Ethics Committee of the Research Division of the Children's Hospital of Chongqing Medical University. Written informed consent to participate in this study was provided by the participants' legal guardian/next of kin.

## Author contributions

XiaohL: conceptualization, planning the methodology, and writing—review and editing. SL, DH, and LZ: investigation and supervision. FT, YF, YR, and XP: validation and obtaining resources. JT, XiaoyL, XinggL, and GH: writing—original draft and writing—review and editing. All authors critically reviewed and approved the final draft of the manuscript.

## Funding

This work was supported by the Basic Research Project of Key Laboratory of the Ministry of Education of China in 2021 (GBRP-202106), the Major Health Project of Chongqing Science and Technology Bureau (CSTC2021jscx-gksb-N0001), the Research and Innovation Team of Chongqing Medical University (W0088), the Joint Medical Research Project of Chongqing Municipal Health Commission and Chongqing Science and Technology Bureau (2020MSXM062), the National Key Research and Development Project (2017YFC0211705), the Education Commission of Chongqing Municipality (KJQN201900443), and the National Natural Science Foundation of China (82003521). The funders had no role in the study design, data collection and analysis, the decision to publish, or the preparation of the manuscript.

## Conflict of interest

The authors declare that the research was conducted in the absence of any commercial or financial relationships that could be construed as a potential conflict of interest.

## Publisher's note

All claims expressed in this article are solely those of the authors and do not necessarily represent those of their affiliated organizations, or those of the publisher, the editors and the reviewers. Any product that may be evaluated in this article, or claim that may be made by its manufacturer, is not guaranteed or endorsed by the publisher.
